# *Pollutimonas rozaliensis* sp. nov., a novel bacterium isolated from gold ore of the active Rozalia mine (Central Europe, Slovakia)

**DOI:** 10.1007/s00203-026-04852-3

**Published:** 2026-03-26

**Authors:** Lea Nosalova, Sona Brestovicova, Jana Kiskova, Tamas Felfoldi, Anna Alexovic Matiasova, Lenka Malinicova, Mariana Kolesarova, Peter Pristas

**Affiliations:** 1 Department of Microbiology, Faculty of Science, Institute of Biology and Ecology, Pavol Jozef Safarik University in Kosice, Srobarova 2, 041 54 Kosice, Slovakia; 2 Present Address: Sorbonne Université, Université Paris Cité, Univ Paris Est Créteil, CNRS, IRD, INRAE, Institut d’écologie et des sciences de l’environnement de Paris, IEES Paris, 75005 Paris, France; 3 Institute of Aquatic Ecology, HUN-REN Centre for Ecological Research, 29 Karolina Str., 1113 Budapest, Hungary; 4 Department of Cell Biology, Faculty of Science, Institute of Biology and Ecology, Pavol Jozef Safarik University in Kosice, Srobarova 2, 041 54 Kosice, Slovakia; 5 Institute of Animal Physiology, Centre of Biosciences, Slovak Academy of Sciences, Soltesovej 4-6, 040 01 Kosice, Slovakia

**Keywords:** *Pollutimonas*, *Pusillimonas*, Gold mine, Heavy-metal resistance

## Abstract

Strain H1-120^T^ was isolated from a heap of mined rock at the Rozalia gold mine (Slovakia) during a study of heavy-metal-resistant microbiota. The cells are Gram-negative, oxidase- and catalase-positive, with optimal growth at 35 °C, pH 6.5–7.5, and 0.5% (*w/v*) NaCl. Phylogenetic analysis based on 16S rRNA gene sequences, together with phylogenomic analysis, placed strain H1-120^T^ within the *Pollutimonas* genus, with *Pollutimonas subterranea* (98.2%), *Pollutimonas bauzanensis* (98.0%), and *Pollutimonas thiosulfatoxidans* (98.0%) as the closest related type strains. Digital DNA–DNA hybridisation, average nucleotide identity, and average amino acid identity values between strain H1-120^T^ and its closely related type strains of the genera *Pollutimonas, Pusillimonas, Parapusillimonas, Allopusillimonas, Mesopusillimonas, Neopusillimonas, Eoetvoesiella* and *Candidimonas* ranged from 19.4% to 22.9%, 71.53% to 80.6%, and 65.47% to 78.72%, respectively. The predominant respiratory quinones were Q8 (98.0%), Q9 (1.2%), and Q7 (0.8%). The major fatty acids were C_16:0_, C_18:1_ *ω*7c, and C_17:0_ cyclo, together with summed feature 2 (iso-C_16:1_ I/C_14:0_ 3-OH and/or C_12:0_ ALDE) and summed feature 3 (C_16:1_ *ω*7c and/or C_16:1_ *ω*6c). The polar lipid profile consisted of phosphatidylglycerol, diphosphatidylglycerol, and phosphatidylethanolamine, together with unidentified aminophospholipids. The genome of strain H1-120^T^ has a G + C content of 59.9 mol% and a size of 4.6 Mb. Based on the 16S rRNA gene phylogeny, genomic data, and phenotypic characteristics of strain H1-120^T^, a novel species with the name *Pollutimonas rozaliensis* sp. nov., is proposed. The type strain is H1-120^T^ (= DSM 118324^T^ = CCM 9437^T^).

**Supplementary Information:**

The online version contains supplementary material available at 10.1007/s00203-026-04852-3.

## Introduction

Despite their extreme conditions, metal-rich deep-subsurface terrestrial environments harbour rich and diverse microbial communities, which remain difficult to isolate, mainly because of insufficient cultivation methods (Nema et al. 2019). The microbiota of subsurface environments is crucial for global biogeochemical cycles (Soares et al. 2023). Moreover, their adaptation to metal-contaminated areas may be of interest for biotechnological applications (Parades-Aguilar et al. 2024). Recent studies on the microbiota of radionuclide-contaminated groundwater led to the isolation of two novel species, initially affiliated with the *Pusillimonas* genus (Grouzdev et al. 2018), followed by reclassification and delineation of novel genera (Babich et al. 2023). At the same time, certain members of the *Pusillimonas* and *Candidimonas* genera were transferred to the *Pollutimonas* genus. At the time of writing, the *Pollutimonas* genus comprises the following validly published species (Oren and Göker 2023): *Pollutimonas subterranea* (type species) and *Pollutimonas nitritireducens* (Babich et al. 2023), *Pollutimonas bauzanensis* (basonym *Candidimonas bauzanensis,* Zhang et al. 2012), *Pollutimonas harenae* (basonym *Pusillimonas harenae,* Park et al. 2011), and *Pollutimonas thiosulfatoxidans* (basonym *Pusillimonas thiosulfatoxidans,* Koh et al. 2019). Representatives of the *Pollutimonas* genus possess isoprenoid Q-8 as the major respiratory quinone; diphosphatidylglycerol (DPG), phosphatidylethanolamine (PE), phosphatidylmethylethanolamine (PME), phosphatidylglycerol (PG), and unidentified aminophospholipids as the major polar lipids; and C_16:0_, C_16:1_ *ω*7c, C_18:1_ *ω*7c, and C_17:0_ cyclo as the dominant fatty acids. Members of the genus are Gram-negative, motile, and mostly neutrophilic. They have been isolated from various habitats, including beach sand, activated sludge, industrial soil contaminated with heavy crude oil and heavy metals, and industrial reservoirs for liquid radioactive waste (Babich et al. 2023). The diversity of isolation sources reflects their ability to adapt to a range of environments, including contaminated ones. In general, bacterial species related to the *Pollutimonas* genus can degrade various pollutants and utilise different compounds as energy sources (Stolz et al. 2005).

During the study of the microbiota of one of the last active gold mines in Central Europe (Nosalova et al. 2021), a novel bacterial strain was isolated, that showed the highest similarity to the genus *Pollutimonas*. In this study, we present the detailed characterisation of the novel strain H1-120^T^.

## Material and methods

### Isolation and cultivation conditions

The H1-120^T^ bacterial strain was isolated from a heap of mined rock accumulated during mining in the Rozalia gold mine (48.4545371 N, 18.7800680 E) (Nosalova et al. 2021). Briefly, approximately 50 g of ore material was sampled aseptically after removing 10 cm of the top layer, and transported to the laboratory in sterile zip-lock bags on ice. The sampled material was processed within 24 h. One gram of sample material was mixed with 10 mL of sterile phosphate-buffered saline supplemented with Tween 80 (250 µL/500 mL phosphate-buffered saline), serially diluted, and aliquots (50 μL) were spread onto NA2 medium (Nutrient Agar no. 2; HiMedia, India). After overnight aerobic cultivation at laboratory temperature (22 °C, 16 h), individual colonies were picked and purified using the streak-plate procedure. Purity of the isolated strain was verified by light microscopy and 16S rRNA gene sequencing. The new strain was stored in glycerol stocks at -70 °C for future analyses and deposited at the Leibniz-Institut DSMZ—German Collection of Microorganisms and Cell Cultures GmbH in Germany and at the Czech Collection of Microorganisms (CCM) under accession numbers DSM 118324^T^ and CCM 9437^T^, respectively.

### Phylogenetic analysis based on the 16S rRNA gene and genome sequencing

To ascertain the phylogenetic position of the H1-120^T^ strain, a nearly complete 16S rRNA gene amplicon sequence was obtained using the fD1 (5′-AGAGTTTGATCCTGGCTCAG-3′) and rP2 (5′-ACGGCTACCTTGTTACGACTT-3′) primer pair (Weisburg et al. 1991) and the TaqCore kit/high yield (Jena Bioscience, Germany), as described previously (Nosalova et al. 2021). Thereafter, the 16S rRNA gene PCR amplicon was cloned using the CloneJET PCR Cloning Kit (Thermo Scientific, Germany) and chemically competent cells of *Escherichia coli* DH10B (Thermo Scientific, Germany). The resulting recombinant plasmid was sequenced at Eurofins Genomics (Germany) using the Sanger sequencing method. The obtained sequences were assembled and analysed using BioEdit v7.2.5 (Hall 1999) and deposited in the GenBank database under accession number PP781967.

Next, 16S rRNA gene sequences of closely related type strains were downloaded from the GenBank database and aligned using ClustalW implemented in the MEGA11 software (Tamura et al. 2021). The evolutionary distances were calculated using the Kimura two-parametric model (Kimura 1980), and the evolutionary history was assessed using the maximum-likelihood algorithm with 1000 bootstrap replications.

Whole-genome sequencing of the H1-120^T^ strain was performed at Eurofins Genomics Europe Sequencing GmbH (Köln, Germany) using Illumina NovaSeq 6000 technology with a paired-end strategy (2 × 150 bp) and an S4 PE150 XP kit. The obtained raw sequences were processed using tools implemented in the Unipro UGENE v35.0 cross-platform bioinformatic software (Okonechnikov et al. 2012; Golosova et al. 2014), checked for quality using the FastQC v0.11.9 tool, and trimmed using the Trimmomatic v0.39 tool with a minimum average quality threshold of 20. The filtered reads were then de novo assembled using the SPAdes v3.14.1 tool, and contigs of < 200 bp were excluded from further analyses. The whole-genome sequence of the H1-120^T^ strain was submitted to the GenBank database under accession number JAZHOB000000000.

Phylogenetic inference of the H1-120^T^ strain was assessed using the maximum-likelihood method based on comparison of core gene sets determined by the EasyCGTree pipeline with default parameters (Zhang et al. 2023). The phylogenetic tree in Newick format was generated and visualised using MEGA 11 software (Tamura et al. 2021). The genome of *Burkholderia cepacia* was used as an outgroup.

For comparative genome analysis, the genomes of the closest representatives of the family *Alcaligenaceae* were downloaded from the GenBank database. Digital DNA-DNA hybridisation (dDDH) values were assessed using the Genome-to-Genome Distance Calculator (GGDC) v3.0 (Meier-Kolthoff et al. 2022) based on the recommended formula 2 (identities/HSP length). Average nucleotide identity (ANI) was estimated using the FastANI tool implemented in the KBase platform (Jain et al. 2018; Arkin et al. 2018) and the OrthoANI tool implemented in the EzBioCloud database (Lee et al. 2016; Yoon et al. 2017). Average amino acid identity (AAI) values were calculated using the Newman Lab AAI calculator (https://newman.lycoming.edu/AAI), based on comparisons of amino-acid sequences obtained from the RAST server (Aziz et al. 2008; Overbeek et al. 2014; Brettin et al. 2015).

The draft genome of the H1-120^T^ strain was further annotated using the SEED tool implemented in the RAST annotation server (Aziz et al. 2008; Overbeek et al. 2014; Brettin et al. 2015), EggNOG mapper (Cantalapiedra et al. 2021), the Bakta online tool (Schwengers et al. 2021), and the PGAP tool v6.6 (NCBI Prokaryotic Genome Annotation Pipeline; Tatusova et al. 2016).

### Microscopy and biochemical assays

The colony morphology of the H1-120^T^ strain was examined using a Leica EZ4 D stereo microscope (Leica Microsystems, Singapore) after cultivation on NA2 medium (Nutrient Agar no. 2; HiMedia, India). Gram staining was performed using the Gram Stain Kit™ (BD Biosciences, USA), and cell morphology was inspected using a BA 310 binocular microscope (Motic, Hong Kong). For detailed visualisation of cellular morphology, overnight bacterial culture (OD_600_ 0.6–0.8) was adhered to formvar-coated slot copper grids, washed with distilled water, negatively stained with 2% uranyl acetate (*w/v*), washed again, and air-dried. The samples were then observed using a transmission electron microscope (JEM 1230; JEOL, Japan) at an accelerating voltage of 80 kV.

Conventional microbiological tests were performed to determine basic phenotypic properties. Motility test was performed using the hanging-drop method. Cytochrome *c* oxidase activity was assessed using oxidase strips (Merck KGaA, Germany), catalase activity was tested using 3% (*v/v*) H_2_O_2_ (CentralChem, Slovakia), and the indole test was performed using Kovacs’ Indole Reagent (HiMedia, India). Christensen agar medium (Thermo Fisher Scientific, Netherlands) was used to test urease activity, DNase activity was assessed using DNase agar (Thermo Fisher Scientific, Netherlands), and TSI medium (HiMedia, India) was used to assess H_2_S production. Growth under anaerobic conditions was tested using an anaerobic jar containing Oxoid AnaeroGen 3.5 L (Thermo Fisher Scientific, Netherlands) after 7 days of cultivation on NA2 medium. Nitrate reduction was tested using the Nitrate Reagent Disks Kit (Merck Millipore, Germany) under anoxic conditions on nitrate agar composed of 5 g/L peptone, 3 g/L yeast extract, and 15 g/L agar (all manufactured by Sigma-Aldrich, Germany) and 1 g/L potassium nitrate (CentralChem, Slovakia). For comparative cultivation and biochemical analyses, the type strains *Pollutimonas bauzanensis* DSM 22805^T^ (= BZ59^T^), *Pollutimonas harenae* DSM 25667^T^ (= B201^T^), *Pollutimonas thiosulfatoxidans* KCTC 62737^T^ (= YE3^T^), *Pollutimonas subterranea* KCTC 62615^T^ (= JR1/69–3-13^T^), and *Pollutimonas nitritireducens* KCTC 62614^T^ (= JR1/69–2-13^T^) were obtained from the Leibniz-Institut DSMZ (Germany) and the Korean Collection for Type Cultures (KCTC), respectively. Biochemical and physiological characteristics and enzymatic activities were further tested using API® ZYM, API® 20E, and API® 50CH (bioMérieux, France) kits (Supplementary Table S1). Growth at 5, 10, 15, 20, 25, 30, 35, 40, and 45 °C; the tolerance to salinity (0, 0.5, 1.0, 1.5, 2.0, 2.5, 3.0, 3.5, 4.0, 4.5, and 5.0% of NaCl (*w/v*)); and growth at pH 5.5–8.5 were tested using Nutrient broth (Merck KGaA, Germany). The effect of pH was tested using broth containing appropriate buffers (citric acid, phosphate, and glycine with sodium hydroxide). The latter two tests were carried out at laboratory temperature (25 °C) in triplicate, simultaneously with the reference strains.

Susceptibility to heavy metals was assessed by Timkova et al. (2020) using the agar dilution method (Schumacher et al. 2018) and in accordance with the requirements of The European Committee on Antimicrobial Susceptibility Testing (EUCAST 2018).

### Chemotaxonomic analyses

For the chemotaxonomic analyses, the H1-120^T^ strain was cultivated on NA2 medium (Merck KGaA, Germany) at laboratory temperature. Analysis of respiratory quinones was performed by HPLC–DAD, and cellular fatty acids were analysed using GC–MS on an Agilent 7000D GC–MS system (Agilent Technologies, USA). Polar lipids were analysed using two-dimensional silica thin-layer chromatography, and the polar lipids were visualised using molybdatophosphoric acid, with specific functional groups detected using spray reagents targeting defined functional groups (Tindall et al. 2007). Analyses of polar lipids, fatty acids, and respiratory quinones were carried out by the Identification Service of the DSMZ (Germany).

## Results and discussion

### Phylogeny of the H1-120^T^ strain

**Fig. 1:**
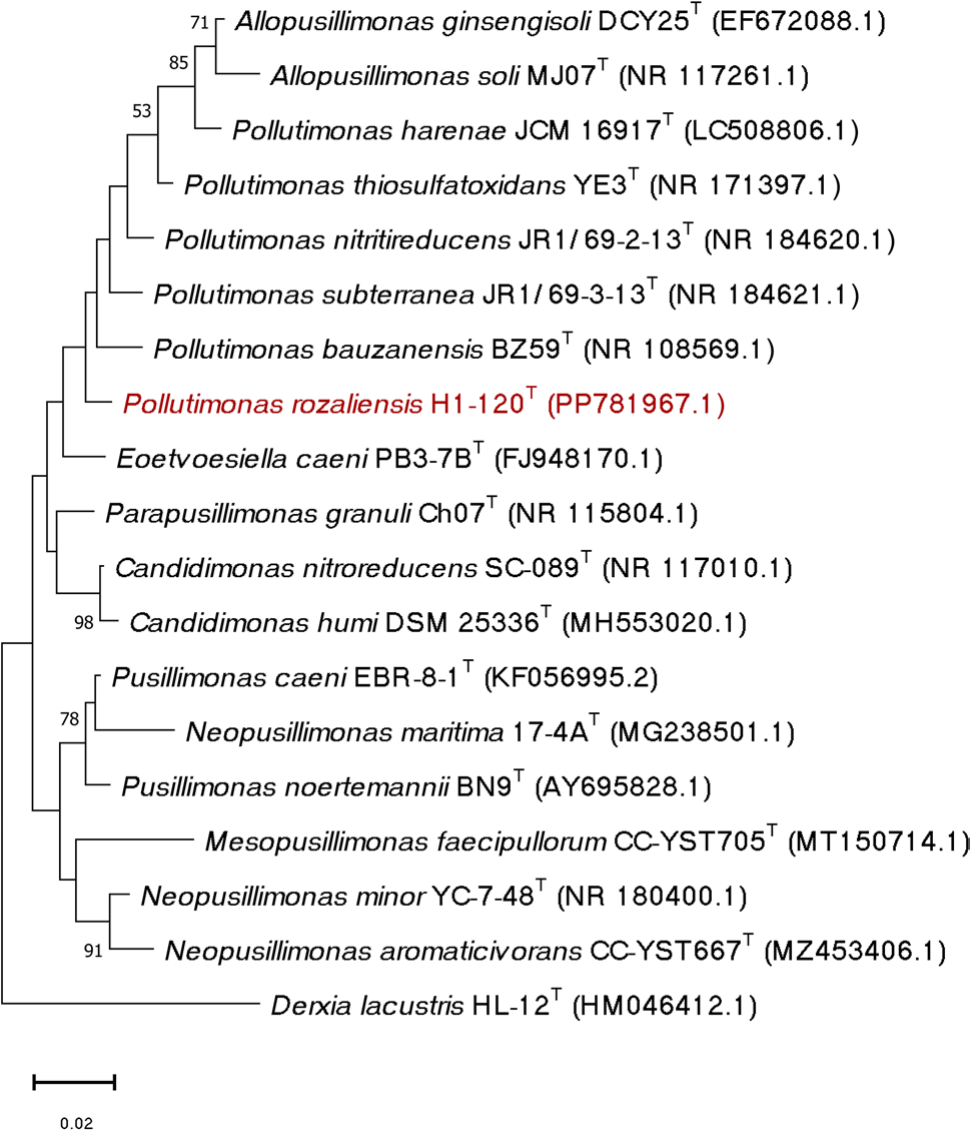
Maximum likelihood phylogenetic tree based on 16S rRNA gene sequences showing the position of strain H1-120^T^ in relation to closely related type strains of the family *Alcaligenaceae*. The scale represents evolutionary distances, in units of the number of base substitutions per site. Bootstrap values of ≥ 50% calculated from 1000 replications are shown at the branch nodes. The 16S rRNA gene sequence of the type strain of *Derxia lacustris* was used as an outgroup

The highest 16S rRNA gene sequence similarities between the H1-120^T^ strain and sequences of type strains database of bacteria and archaea (rRNA/ITS) were observed with *Pollutimonas subterranea* JR1/69–3-13^T^ (98.2%), followed by *Pollutimonas bauzanensis* BZ59^T^ (98.0%) and *Pollutimonas thiosulfatoxidans* YE3^T^ (98.0%). Similarly, the closest relationship with *Pollutimonas subterranea* JR1/69–3-13^T^ (98.4%) was observed when compared with the EzBioCloud database (Yoon et al. 2017). These values are below the generally accepted threshold of 98.7% used to delineate bacterial species (Kim et al. 2014) and imply that the H1-120^T^ strain may represent a novel species within the *Pollutimonas* genus. In the 16S rRNA-gene based phylogenetic tree, the H1-120^T^ strain formed a separate lineage within the *Pollutimonas* genus branch (Fig. 1), although with low bootstrap support.

The genome sequence of the H1-120^T^ strain consisted of 403 scaffolds with a total length of 4,627,112 bp and an overall G + C content of 59.9 mol%. The assembly retrieved 400 contigs, with an N50 value of 363.6 kb and an L50 value of 5. Based on CheckM analysis (v1.2.3) provided by the NCBI database, contamination was estimated at 1.85% and completeness at 99.39%. The genome contains approximately 4600 CDSs. The dDDH values against the analysed genomes (selected based on 16S rRNA gene similarity) were in the range from 19.4% to 22.9%, which is significantly lower than the widely accepted threshold of 70% used for species delineation (Meier-Kolthoff et al. 2013). ANI values assessed using FastANI ranged from 77.31% to 80.6%, and from 71.53% to 78.4% according to OrthoANI, confirming that the studied strain represents a new species because these values are below the suggested threshold of 95–96% (Jain et al. 2018). The observed AAI values ranged from 65.47% to 78.72%, which also fall below the threshold for species delineation. These values confirmed the affiliation of the studied isolate with the *Pollutimonas* genus. The obtained dDDH, ANI, and AAI values are summarised in Supplementary Table S2.

**Fig. 2:**
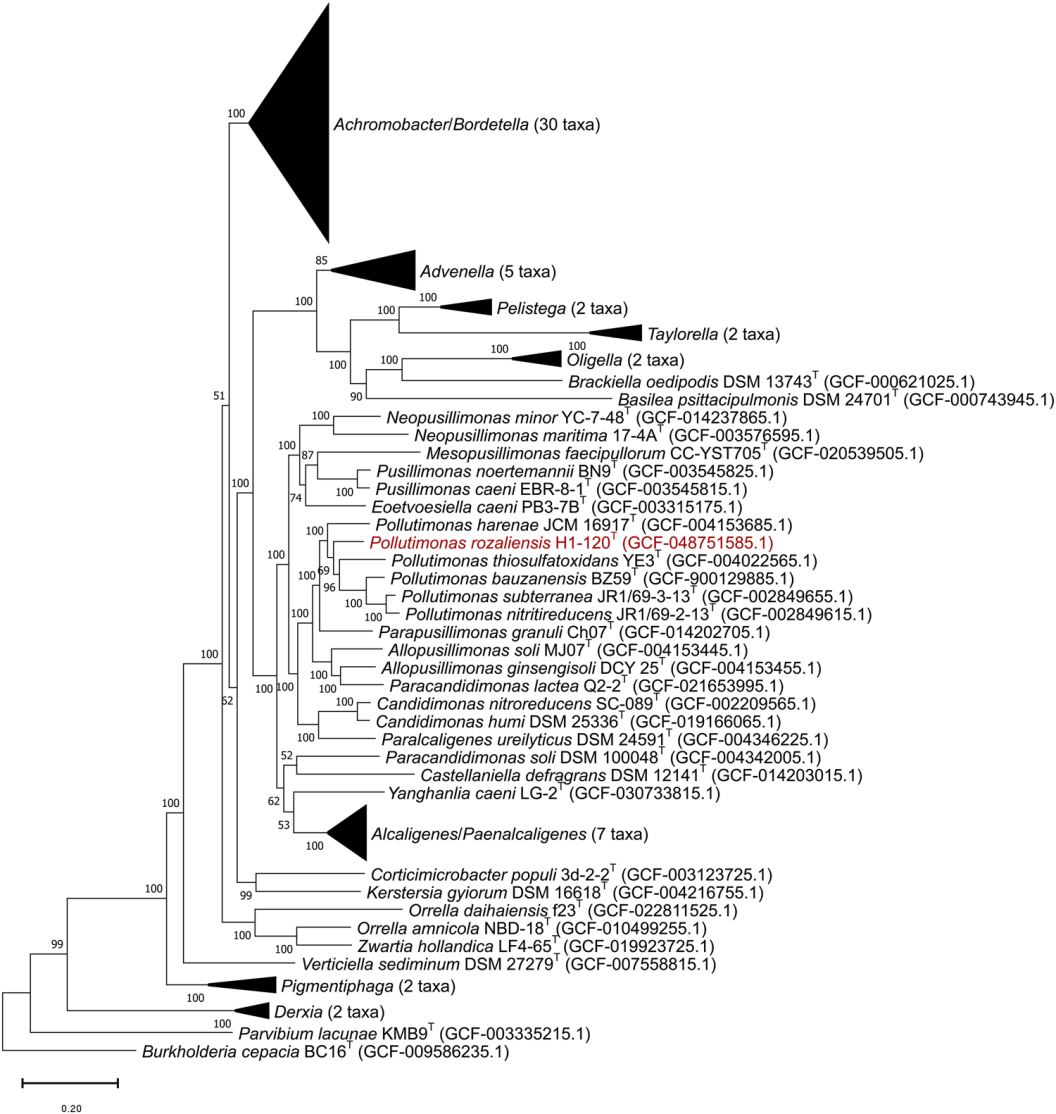
Maximum likelihood phylogenetic tree showing the position of strain H1-120^T^ among type strains of the family *Alcaligenaceae*. The tree was reconstructed using the genome of strain H1-120^T^ and genomes of the type strains of the family *Alcaligenaceae* available in the GenBank database, with 1000 bootstrap replications. The genome of *Burkholderia cepacia* was used as an outgroup

Consistent with the results of the 16S rRNA gene sequence analysis, which showed the closest phylogenetic relatedness of the H1-120^T^ strain to *Pollutimonas subterranea*, comparative genomic analysis also indicated the closest relationship with the *Pollutimonas subterranea, Pollutimonas nitritireducens,* and *Pollutimonas bauzanensis* cluster. Moreover, based on the phylogenomic tree, the H1-120^T^ strain formed a separate branch within the *Pollutimonas* cluster (Fig. 2). Both approaches confirmed that the H1-120^T^ strain belongs to the *Pollutimonas* genus.

Further annotation analysis using the SEED tool showed genome characteristics similar to those of other representatives of the *Pollutimonas* genus. In particular, the genome size was approximately 4.6 Mb, with around 4600 coding sequences and about 50 RNA-encoding genes (Supplementary Table S3). Interestingly, while *Pollutimonas* sp. strain M17 showed the highest similarity in all genomic analyses (dDDH/ANI/AAI values of 31.6/86.5–86.7/90.5), it has a significantly smaller genome (3.9 Mbp versus 4.6 Mbp). This difference may be due to the abundance of heavy-metal resistance genes present in the H1-120^T^ strain but absent from the M17 strain genome, as shown by comparison of metabolic reconstructions of the genomes assessed using the SEED tool. In particular, genes related to copper, cobalt-zinc-cadmium, chromium and mercury resistance were found in the genome of the H1-120^T^ strain but not in the M17 strain. The higher abundance of heavy-metal resistance genes may reflect adaptation of the strain to the harsh environment of mined ore from the gold mine heap. Therefore, the genetic background of heavy-metal resistance and stress response was further investigated. Heavy-metal resistance genes were annotated using the PGAP tool and compared with the GenBank nr database using the BlastX tool. The best hits identified in the heavy-metal resistance gene analysis are summarised in Supplementary Table S4.

### Cell morphology and biochemistry

**Fig. 3:**
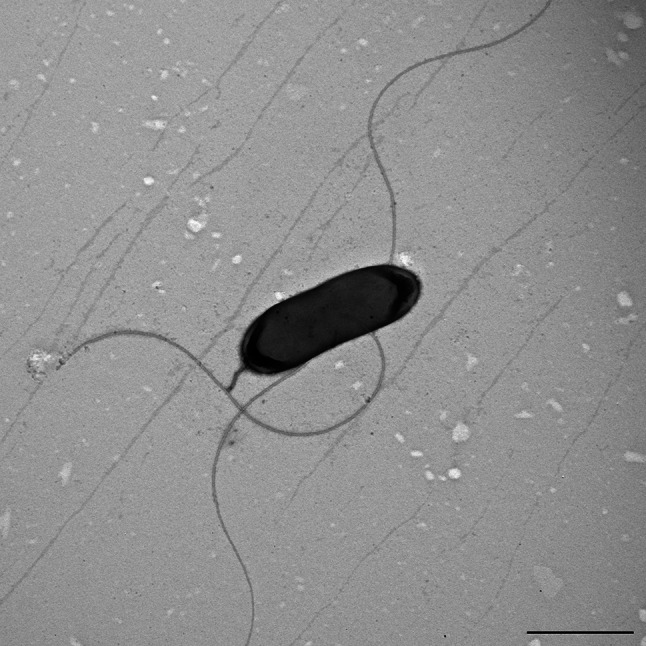
Electron micrograph of a negatively stained cell of strain H1-120^T^; scale bar 2 μm

When grown on NA2 medium, the colonies were creamy and smooth with entire edges. Light microscopy and transmission electron microscopy observations showed that the cells were Gram-negative short rods. The cells had an elliptical shape with polar flagella (mostly one to two) (Fig. 3). Motility was confirmed using the hanging-drop method under a light microscope.

Strain H1-120^T^ grew within a temperature range of 15–40 °C, with the optimum observed at 35 °C. The growth occurred at NaCl concentrations ranging from 0 to 4.0%, with optimal growth at 0.5% NaCl (*w/v*). Growth was observed at pH 6.0–8.5, with an optimum between pH 6.5 and 7.5. According to API® ZYM results, the strain was positive for esterase (C4), leucine arylamidase, and alkaline phosphatase and weakly positive for esterase lipase, trypsin, acid phosphatase, naphthol-AS-BI-phosphohydrolase, α-galactosidase, β-galactosidase, β-glucuronidase, β-glucosidase, N-acetyl-β-glucosaminidase, α-mannosidase, and α-fucosidase. The strain showed a positive result for nitrate reduction under anaerobic conditions. Among the API® 50CH test, positive reactions were observed for D-galactose and D-glucose, while weakly positive reaction was also observed for D-lactose. According to API® 20E results, the strain was able to utilise D-sucrose and citrate and showed positive reactions for arginine dihydrolase, lysine decarboxylase, and ornithine decarboxylase. A positive reaction was also observed for urease test. All results on morphological, biochemical, and physiological characteristics are provided in the species description, and differential characteristics are listed in Table 1.

**Table 1:** The main differential characteristics of strain H1-120^T^ compared with the type strains of other species within the *Pollutimonas* genus

	1	2	3	4	5	6
Cell size [μm]	0.5–0.7 × 1.2–2.1	0.7–1.3 × 0.9- 2.1	0.8–1.2 × 1.5–2.1	0.7–1.2 × 2.0- 2.8	0.7–0.9 × 1.2- 1.9	0.5–0.7 × 0.6- 0.9
Colony pigmentation	Creamy	Creamy	Creamy	Pale yellow	Creamy white	White
*Growth*						
pH range (optimum)	6–8.5 (6.5–7.5)	6.0–8.5 (6.5–7.7)	6.0–8.5 (6.5–7.6)	5.5–9.0 (7.0)	6.0–8.0 (7.4–7.8)	5.0–9.0 (7.0)
Temperature range (optimum) [°C]	15–40 (35)	10–33 (28)	5–33 (28)	10–40 (30)	1–37 (25–30)	15–45 (30)
NaCl range (optimum) [%, *w/v*]	0–4.0 (0.5)	0–5.0 (1.0–3.0)	0–3.0 (0.5–1.0)	1.0–8.0 (1.0)	0–3.0	0–6.0 (0–3.0)
Nitrate reduction	+	+	-	-	+	-
Enzyme activities*						
API® ZYM						
Alkaline phosphatase	+	-	w	+	-	w
Valine arylamidase	-	-	-	+	-	+
Cystine arylamidase	-	-	-	-	-	+
α-chymotrypsin	-	-	-	-	-	+
API® 50CH						
D-Galactose	+	-	-	+	+	-
D-Glucose	+	-	w	+	+	-
API® 20E						
Lysine decarboxylase	+	-	-	-	-	-
Ornithine decarboxylase	+	-	-	-	-	-
Citrate utilization	+	-	w	w	-	-
Urease	+	-	+	-	-	-
D-sucrose utilization	+	-	+	-	-	-
Major polar lipids**	DPG, PE, PG, PL, AL, L	DPG, PE, PG, PME, APL1, APL3	DPG, PE, PG, PME, APL1, APL3	DPG, PG, PE	DPG, PE, PG, PME, AL, APL, L1, L2	DPG, PE, PG, PL, AL1, AL2, AL3
Major cellular fatty acids***	C_12:0_, C_16:0_, C_17:0_ cyclo, C_18:1_ *ω7c*, SF2, SF3	C_16:0_, C_16:1_ *ω7c*, C_17:0_ cyclo, C_18:1_ *ω7c*	C_16:0_, C_16:1_ *ω7c*, C_17:0_ cyclo, C_18:1_ *ω7c*	C_12:0_, C_16:0_, C_17:0_ cyclo, SF2	C_16:0_, C_17:0_ cyclo, C_18:1_ *ω7c*, SF3	C_12:0_, C_16:0_, C_17:0_ cyclo, C_19:0_ *ω8c*, SF2
Genomic DNA G + C content [mol%]	59.9	57.9	57.2	59.3	62.2	56.3

Data for strain H1-120ᵀ were obtained in the present study

Data for the reference strains were taken from previously published descriptions unless otherwise indicated. Growth preferences and API® 50CH and API® 20E tests were determined in this study

Strains: 1, *Pollutimonas rozaliensis* H1-120^T^ (data from this study); 2, *Pollutimonas subterranea* JR1/69–3-13^T^ (Babich et al. 2023); 3, *Pollutimonas nitritireducens* JR1/69–2-13^T^ (Babich et al. 2023); 4, *Pollutimonas thiosulfatoxidans* YE3^T^ (Koh et al. 2019); 5, *Pollutimonas bauzanensis* BZ59^T^ (Zhang et al. 2012), 6, *Pollutimonas harenae* B201^T^ (Park et al. 2011; Li et al. 2020)

*For API® ZYM, API® 50CH and API® 20E results were evaluated as + positive; – negative; w weakly positive

**DPG, diphosphatidylglycerol; PE, phosphatidylethanolamine; PG, phosphatidylglycerol; PI, phosphatidylinositol; PME, phosphatidyl-N-methyl-ethanolamine; PL, unidentified phospholipid; GL, unidentified glycolipids; AL, unidentified aminolipid; APL, unidentified aminophospholipid; L, unidentified lipid; PC, phosphatidylcholine

***SF 2, summed feature 2 (iso-C_16:1_ I/C_14:0_ 3-OH and/or C_12:0_ ALDE); SF 3, summed feature 3 (C_16:1_ *ω7c* and/or C_16:1_ *ω6c*)

The analysed genome contains multiple heavy-metal resistance genes (Supplementary Table S4), and susceptibility testing showed relatively high tolerance to selected heavy metals. The highest metal concentrations at which the strain showed growth were observed for zinc (250 mg/L), copper (250 mg/L), nickel (500 mg/L), and lead (2000 mg/L). The H1-120^T^ strain was isolated from a heap at the Rozalia gold mine, where mining activities mainly focused on copper, lead, and zinc, as well as gold and silver. The analysed samples of mined material accumulated in the heap contained high levels of heavy metals (Zn 2481 mg/kg, Fe 34,123 mg/kg, Au 12 mg/kg, Pb 1093 mg/kg, Ni 25 mg/kg) (Sejkora et al. 2015; Nosalova et al. 2021). Environments with high heavy metal contents are a natural source of heavy-metal-resistant microbiota, with tolerance significantly higher than that observed in to non-contaminated environments. Therefore, the observed tolerance of strain H1-120^T^ to selected heavy metals is consistent with that reported for microbiota from contaminated sites adapted to harsh environmental conditions (Sinegani and Younessi 2017; Pal et al. 2022).

### Chemotaxonomy

The main respiratory quinones of the H1-120^T^ strain were Q8 (98%), followed by Q9 (1.2%) and Q7 (0.8%), which agrees with literature data, indicating that isoprenoid Q8 represents the main respiratory quinone of the *Pollutimonas* genus (Babich et al. 2023). Moreover, Q9 quinone was also identified as the minor part in the species *Pollutimonas subterranea* and *Pollutimonas nitritireducens* (Babich et al. 2023). The polar lipid pattern was predominantly composed of diphosphatidylglycerol, phosphatidylethanolamine, phosphatidylglycerol, an unidentified aminolipid, an unidentified phospholipid, and an unidentified lipid (Supplementary Figure S1); which are the main lipids detected among the representatives of the *Pollutimonas* genus (Babich et al. 2023). In addition to the polar lipids here mentioned, and identified in the H1-120^T^ strain, other polar lipids such as phosphatidyl-N-methyl-ethanolamine and an unidentified aminophospholipid have been reported in *Pollutimonas* representatives (Zhang et al. 2012; Babich et al. 2023). The major cellular fatty acids identified in the strain H1-120^T^ were C_16:0_ (23.9%), C_18:1_ *ω*7c (16.6%), C_17:0_ cyclo (10.6%), and C_12:0_ (5.4%) (Supplementary Table S5). Fatty acid profiles of the *Pollutimonas* genus typically contain C_16:0_ in high amounts, while C_18:1_ *ω*7c and C_17:0_ cyclo occur in varying proportions. Several differences in the fatty acid profiles between the H1-120^T^ strain and the closest representatives of the *Pollutimonas* genus were observed. For example, C_16:1_ *ω*7c accounted for approximately one-third of the identified fatty acids in the phylogenetically closest species *Pollutimonas subterranea* JR1/69–3-13^T^ and *Pollutimonas nitritireducens* JR1/69–2-13^T^ but was not detected in the H1-120^T^ strain (Babich et al. 2023) (Supplementary Table S5). These species also differed in the very low (< 2%) amounts of C_12:0_ detected, whereas strain H1-120^T^*, Pollutimonas thiosulfatoxidans* YE3^T^, and *Pollutimonas harenae* B201^T^ showed similar and higher amounts of C_12:0_ in the range of 5.4–7.9% (Park et al. 2011; Koh et al. 2019).

Based on the polyphasic analysis presented here, we conclude that strain H1-120^T^ represents a novel species of the *Pollutimonas* genus, for which the name *Pollutimonas rozaliensis* sp. nov. is proposed.

### Description of *Pollutimonas rozaliensis* sp. nov.

*Pollutimonas rozaliensis* (ro.za.li.en′sis. N.L. fem. adj. *rozaliensis*, referring to the Rozalia mine, village Hodruša-Hámre, Slovakia, where the type strain was isolated).

Cells are Gram-negative, facultatively anaerobic, non-spore-forming, motile rods measuring 0.5–0.7 × 1.2–2.1 μm, motile by polar flagella. Growth occurs at 15–40 °C (optimum 35 °C), at pH 6.0–8.5 (optimum 6.5–7.5), and in the presence of 0–4.0% (*w/v*) NaCl (optimum 0.5%). Colonies cultivated on NA2 medium are creamy, convex, and round with entire margins. Cells are catalase- and oxidase-positive and according to API® 20E, positive for lysine decarboxylase and ornithine decarboxylase. The strain is negative for gelatin hydrolysis, and production of indole, acetoin, and H_2_S, and positive for urease. The major cellular polar lipids are phosphatidylglycerol, diphosphatidylglycerol, and phosphatidylethanolamine, together with unidentified aminophospholipids. The major cellular fatty acids (> 5% of total fatty acids) are C_12:0_, C_16:0_, C_17:0_ cyclo, C_18:1_ *ω*7c, summed feature 2 (iso-C_16:1_ I/C_14:0_ 3-OH and/or C_12:0_ ALDE), and summed feature 3 (C_16:1_ *ω*7c and/or C_16:1_ *ω*6c).

The type strain is H1-120^T^ (= DSM 118324^T^ = CCM 9437^T^), isolated from ore material mined from the Rozalia gold mine in Central Europe (Slovakia). The genome size of the type strain is 4.6 Mb with an overall G + C content of 59.9 mol%. The genome sequence has been deposited in GenBank under accession number JAZHOB000000000, and the 16S rRNA gene sequence under accession number PP781967.

## Supplementary Information

Below is the link to the electronic supplementary material.

Supplementary Material 1

## Data Availability

The 16 S rRNA gene sequence and the whole-genome sequence analysed during the study are available in the GenBank database under the accession numbers PP781967 and JAZHOB000000000, respectively.
