# Supplementary material for: Pollutimonas rozaliensis sp. nov., a novel bacterium isolated from gold ore of the active Rozalia mine (Central Europe, Slovakia)
Source: Arch Microbiol. 2026 Mar 26;208(6):287. doi: 10.1007/s00203-026-04852-3 (PMC13021757; doi:10.1007/s00203-026-04852-3)
Supplement: Supplementary file 1 — Supplementary Material 1 [file 203_2026_4852_MOESM1_ESM.pdf]

Supplementary Table S1 The results of the API® 20E, API® 50CH and API® ZYM tests (bioMérieux, France) of strain H1-120^T compared to type strains of the genus *Pollutimonas*. Results were evaluated as + positive; – negative; w weakly positive

Strains: 1, *Pollutimonas rozaliensis* H1-120^T (this study); 2, *Pollutimonas subterranea* JR1/69-3-13^T; 3, *Pollutimonas nitritireducens* JR1/69-2-13^T; 4, *Pollutimonas thiosulfatoxidans* YE3^T; 5, *Pollutimonas bauzanensis* BZ59^T; 6, *Pollutimonas harenae* B201^T. Data were taken from this study unless otherwise stated

	1	2	3	4	5	6
API® 20E						
ß-galactosidase	-	-	+	-	-	-
Arginine dihydrolase	w	-	w	-	-	-
Lysine decarboxylase	+	-	-	-	-	-
Ornithine decarboxylase	+	-	-	-	-	-
Citrate utilization	+	-	w	w	-	-
H_2S production	-	-	-	-	-	-
Urease	+	-	+	-	-	-
Tryptophane deaminase	-	-	-	+	-	-
Indole production	-	-	-	-	-	-
acetoin production	-	-	-	-	+	w
Gelatinase	-	-	+	-	-	-
D-glucose utilization	-	-	+	-	+	-
D-mannitol utilization	-	-	w	-	-	-
Inositol utilization	-	-	-	-	-	-
D-sorbitol utilization	-	-	-	-	-	-
L-rhamnose utilization	-	-	-	-	-	-
D-sucrose utilization	+	-	+	-	-	-
D-melibiose utilization	-	-	-	-	-	-
Amygdalin utilization	-	-	-	-	-	-
L-arabinose utilization	-	-	-	-	+	-
NO_2 production	-	-	-	-	+	-
API® 50CH						
Glycerol	-	-	-	+	w	-
Erythritol	-	-	-	-	-	-
D-Arabinose	-	-	-	+	+	-
L-Arabinose	-	-	-	+	+	-
D-Ribose	-	-	-	+	+	-
D-Xylose	-	-	-	+	+	-
L-Xylose	-	-	-	+	+	-
D-Adonitol	-	-	-	-	-	-

Methyl-ß-D-xylopyranoside	-	-	-	-	-	-
D-Galactose	+	-	-	+	+	-
D-Glucose	+	-	w	+	+	-
D-Fructose	-	-	-	-	-	-
D-Mannose	-	-	-	+	+	-
L-Sorbose	-	-	-	-	-	-
L-Rhamnose	-	-	-	w	+	-
Dulcitol	-	-	-	-	-	-
Inositol	-	-	-	-	-	-
D-Mannitol	-	-	-	-	-	-
D-Sorbitol	-	-	-	-	-	-
Methyl-α-D-mannopyranoside	-	-	-	-	-	-
Methyl-α-D-glucopyranoside	-	-	-	-	-	-
Amygdalin	-	-	-	-	-	-
Arbutin	-	-	-	-	-	-
Esculin	-	-	-	-	-	-
Salicin	-	-	-	-	-	-
D-Cellobiose	-	-	-	-	-	-
D-Maltose	-	-	-	-	-	-
D-Lactose	-	-	-	-	-	-
D-Melibiose	w	-	-	-	-	-
D-Saccharose	-	-	-	-	-	-
D-Trehalose	-	-	-	-	-	-
Inulin	-	-	-	-	-	-
D-Melezitose	-	-	-	-	-	-
D-Raffinose	-	-	-	-	-	-
Starch	-	-	-	-	-	-
Glycogen	-	-	-	-	-	-
Xylitol	-	-	-	-	-	-
Gentiobiose	-	-	-	-	-	-
D-Turanose	-	-	-	-	-	-
D-Lyxose	-	-	-	-	-	-
D-Tagatose	-	-	-	-	-	-
D-Fucose	-	-	-	-	-	-
D-Arabitol	-	-	-	w	+	-
L-Arabitol	-	-	-	-	-	-
Potassium gluconate	-	-	-	-	-	-
Potassium 2-ketogluconate	-	-	-	-	-	-
Potassium 5-ketogluconate	-	-	-	-	-	-
API® ZYM*						
Alkaline phospatase	+	w	-	-	w	+
Esterase	+	+	+	+	+	+
Esterase lipase	w	+	+	w	+	+
Lipase	-	-	-	-	-	-
Leucine arylamidase	+	+	w	w	+	+
Valine arylamidase	-	-	-	-	+	+
Cystine arylamidase	-	-	-	-	+	-
Trypsin	w	-	-	-	w	-
α-chymotrypsin	-	-	-	-	+	-

Acidic phosphatase	w	+	w	w	+	+
Naphtol-AS-BI-phosphohydrolase	w	+	+	+	+	+
α-galactosidase	w	-	-	-	-	-
β-galactosidase	w	-	-	-	-	-
β- glucuronidase	w	-	-	-	-	-
α- glucosidase	-	-	-	-	-	-
β- glucosidase	w	-	-	-	-	-
N-acetyl-β-glucosaminidase	w	-	-	-	-	-
α-mannosidase	w	-	-	-	-	-
α-fucosidase	w	-	-	-	-	-

*Data for columns 2 and 3 were obtained from Babich et al. (2023), for column 4 from Koh et al. (2019); for column 5 from Zhang et al. (2012), and for column 6 from Park et al. (2011) and Li et al. (2020)

Supplementary Table S2 *In silico* DNA-DNA hybridisation (dDDH), average nucleotide identity (ANI), and average amino acid identity (AAI) values of strain H1-120^T, compared to genomes of genus *Pollutimonas* and neighboring taxa

	dDDH	ANI (FastANI)	ANI (OrthoANI)	AAI (Newman Lab)
Pollutimonas subterranea JR1/69-3-13^T (PDNW01000001)	21.6	80.3	77.11	77.6
Pollutimonas nitritireducens JR1/69-2-13^T (PDNV01000001)	20.9	79.7	76.1	77.2
Pollutimonas thiosulfatoxidans YE3^T (CP022987)	21.5	80.6	77.7	78.1
Pollutimonas bauzanensis BZ59^T (FQXE01000001)	22.0	80.4	78.4	77.1
Pollutimonas harenae B201^T (SDQD00000000)	20.3	79.17	76.40	78.72
Parapusillimonas granuli DSM 18079^T (JACHHM000000000)	21.4	80.49	77.65	74.87
Allopusillimonas ginsengisoli KCTC 22046^T (SDQE00000000)	22.6	79.91	75.98	74.54
Allopusillimonas soli KCTC 22455^T (SDQC00000000)	20.1	78.76	74.97	72.59
Eoetvoesiella caeni NCAIM B.02512^T (JBHUNU000000000)	21.5	79.4	75.66	71.26
Neopusillimonas minor YC-7-48^T (JACJUU000000000)	20.7	78.57	73.54	68.1
Pusillimonas noertemannii DSM 10065^T (PDUX00000000)	20.4	79.11	75.37	68.49
Pusillimonas caeni KCTC 42353^T (PDUW00000000)	22.9	80.42	76.63	69.77
Mesopusillimonas faecipullorum CC-YST705^T (JACDXW000000000)	20.5	77.99	72.26	65.47
Neopusillimoas aromaticivorans CC-YST667^T (CP128233)	19.4	77.67	72.79	68.76
Neopusillimonas maritima 17-4A^T (NQOU00000000)	20.5	77.31	71.53	65.79
Candidimonas humi DSM 25336^T (JAHTBN000000000)	21.5	79.10	75.81	69.94
Candidimonas nitroreducens SC-089^T (NJIH00000000)	21.5	79.11	76.16	69.36

Supplementary Table S3 Basic genomic characteristics of strain H1-120^T and representatives of genus *Pollutimonas* and neighboring taxa available in the GenBank database

	GenBank accession number	Genome size [Mb]	G+C content [mol%]	Genes	rRNA genes	tRNA genes	Protein-coding genes	Pseudogenes
H1-120^T	JAZHOB000000000	4.6	59.9	4648	3	45	4528	68
Pollutimonas subterranea JR1/69-3-13^T	PDNW01000001	4.5	58	4270	5	45	4118	98
Pollutimonas nitritireducens JR1/69-2-13^T	PDNV01000001	4.3	57	4122	3	44	3981	90
Pollutimonas thiosulfatoxidans YE3^T	CP022987	3.5	59.5	3411	6	44	3285	72
Pollutimonas bauzanensis BZ59^T	FQXE01000001	5.6	62	5210	5	42	5154	114
Pollutimonas harenae B201^T	SDQD00000000	3.4	56.5	3177	3	44	3099	27
Parapusillimonas granuli DSM 18079^T	JACHHM000000000	4.6	64.5	4376	3	45	4322	126
Allopusillimonas ginsengisoli KCTC 22046^T	SDQE00000000	4.5	58	4314	3	45	4169	93
Allopusillimonas soli KCTC 22455^T	SDQC00000000	4.1	60	3938	3	43	3832	56
Eoetvoesiella caeni NCAIM B.02512^T	JBHUNU000000000	4.8	59	4612	6	46	4848	72
Neopusillimonas minor YC-7-48^T	JACJUU000000000	3.2	57.5	3035	7	44	2928	51
Pusillimonas noertemannii DSM 10065^T	PDUX00000000	4.2	62.5	3928	5	43	3836	40
Pusillimonas caeni KCTC 42353^T	PDUW00000000	4.4	63	4222	3	43	4120	52
Mesopusillimonas faecipullorum CC-YST705^T	JACDXW000000000	3.1	57.5	3014	14	46	2895	55
Neopusillimonas aromaticivorans CC-YST667^T	CP128233	3.3	57	3639	9	42	2613	971
Neopusillimonas maritima 17-4A^T	NQOU00000000	3.3	53.5	3103	3	40	3012	44

Candidimonas humi DSM 25336^T	JAHTBN000000000	4.7	64.5	4237	7	41	4162	23
Candidimonas nitroreducens SC-089^T	NJIH00000000	5.6	63.5	4989	3	42	4870	70

Supplementary Table S4 Genetic determinants of multidrug resistance annotated within the genome of the H1-120^T strain using PGAP tool and compared with the GenBank nr database using the BlastX tool

Category	Gene	Predicted function by PGAP	The highest similarity with nr database	The accession number of the best hit	Amino acid sequence similarity [%]
Arsenic resistance	aioB	Arsenate reductase (azurin) small subunit	Arsenate reductase (azurin) large subunit [*Pollutimonas harenae*]	WP_130037564.1	97.71
			Arsenate reductase (azurin) small subunit [*Pollutimonas subterranea*]	WP_102075887.1	92.05
	arsB	Arsenic transporter protein	Arsenic transporter [*Mesopusillimonas faecipullorum*]	WP_226955362.1	91.82
			Arsenic transporter [*Pollutimonas harenae*]	WP_130037622.1	94.61
			Arsenic resistance protein [*Orrella marina*]	WP_108623208.1	86.24
	arsC	Arsenate reductase ArsC	Arsenate reductase ArsC [*Eoetvoesiella caeni*]	WP_113933172.1	91.19
			Arsenate reductase ArsC [*Pollutimonas subterranea*]	WP_102075858.1	91.19
			Arsenate reductase ArsC [*Pusillimonas* sp. T7-7]	WP_013742166.1	85.55
			Arsenate reductase ArsC [*Pollutimonas harenae*]	WP_130037619.1	90.64
			Arsenate reductase (glutaredoxin) [*Alcaligenaceae*]	WP_129968247.1	92.91
			Arsenate reductase (glutaredoxin) [*Pollutimonas harenae*]	WP_130037574.1	91.49
			Arsenate reductase (glutaredoxin) [*Pollutimonas* sp. M17]	WP_264132379.1	85.09
			Arsenate reductase ArsC [*Eoetvoesiella caeni*]	WP_113933172.1	91.14
			Arsenate reductase ArsC [*Pollutimonas harenae*]	WP_130037619.1	90.59
	arsH	Arsenical resistance protein ArsH	Arsenical resistance protein ArsH [*Pollutimonas subterranea*]	WP_102075854.1	90.76
			Arsenical resistance protein ArsH [*Orrella marina*]	WP_108621349.1	91.67
	arsR	Metalloregulator ArsR/SmtB family transcription factor	Metalloregulator ArsR/SmtB family transcription factor [*Pollutimonas harenae*]	WP_130037617.1	91.07
			Metalloregulator ArsR/SmtB family transcription factor [*Mesopusillimonas faecipullorum*]	WP_226955368.1	91.15
			Metalloregulator ArsR/SmtB family transcription factor [Alcaligenaceae]	WP_118994248.1	100
			Metalloregulator ArsR/SmtB family transcription factor [*Pollutimonas bauzanensis*]	WP_073107547.1	90.2
			Metalloregulator ArsR/SmtB family transcription factor [*Massilia* sp. DJPM01]	WP_229255608.1	98.17
			Metalloregulator ArsR/SmtB family transcription factor [*Herbaspirillum*]	WP_284078246.1	100
Chromium resistance	chrA	Chromate transporter	Chromate transporter [*Pollutimonas* sp. M17]	WP_264130789.1	91.71

			Chromate transporter [*Pollutimonas* sp. M17]	WP_264130788.1	94.86
			Chromate efflux transporter [uncultured *Castellaniella* sp.]	WP_353155094.1	89.09
			Chromate resistance efflux protein ChrA [Pseudomonadota]	WP_033470574.1	100
			Chromate efflux transporter [*Pollutimonas* sp. M17]	WP_264128932.1	81.86
	chrB	Chromate resistance protein ChrB	Chromate resistance protein ChrB domain-containing protein [*Candidatus* Nitrotoga sp. AM1P]	WP_173052553.1	84
			Chromate resistance protein ChrB domain-containing protein [uncultured *Castellaniella* sp.]	WP_173052553.1	75.96
			Chromate resistance protein ChrB domain-containing protein [Pseudomonadota]	WP_173052553.1	100
Copper resistance	copA	Copper resistance protein mutlicopper oxidase	Copper resistance system multicopper oxidase [*Castellaniella* sp. S9]	WP_269495742.1	100
			Copper resistance system multicopper oxidase [*Pollutimonas bauzanensis*]	WP_073110287.1	92.01
	copB	Copper resistance protein B	Copper resistance protein B [*Pollutimonas thiosulfatoxidans*]	WP_164878339.1	100
			Copper resistance protein B [*Eoetvoesiella caeni*]	WP_242341922.1	78.95
	copC	Copper-binding protein	Copper-binding protein [*Pollutimonas subterranea*]	WP_102075251.1	92.37
	copD	CopD family protein	CopD family protein [*Pollutimonas bauzanensis*]	WP_353153019.1	86.23
	copG	CopG family transcriptional regulator	CopG family transcriptional regulator [*Alcaligenes* sp. NLF5-7]	WP_254499624.1	100
Mercury resistance	merA	Mercury(II) reductase	Mercury(II) reductase [Oxalobacteraceae]	WP_012079604.1	100
	merC	Organomercurial transporter MerC	Organomercurial transporter MerC [Bacteria]	WP_000522996.1	100
	merD	Mercury resistance co-regulator MerD	Mercury resistance co-regulator MerD [Oxalobacteraceae]	WP_012079603.1	100
	merE	Broad-spectrum mercury transporter MerE	Broad-spectrum mercury transporter MerE [Oxalobacteraceae]	WP_012079602.1	100
			Broad-spectrum mercury transporter MerE [Bacteria]	WP_005413392.1	100
	merF	Mercury resistance system transport protein MerF	Mercury resistance system transport protein MerF [Pseudomonadota]	WP_000654684.1	100
	merP	Mercury resistance system periplasmic binding protein MerP	Mercury resistance system periplasmic binding protein MerP [*Rhodoferax* sp.]	MDP2192644.1	75.53
			Mercury resistance system periplasmic binding protein MerP [Bacteria]	WP_004178136.1	100
			Mercury resistance system periplasmic binding protein MerP [Pseudomonadota]	WP_000735441.1	100
	merR	MerR family DNA-binding transcriptional regulator	MerR family transcriptional regulator [Betaproteobacteria bacterium]	MDA8129631.1	72.99
			Hg(II)-responsive transcriptional regulator [*Pararhodobacter* sp.]	WP_323034576.1	77.78
			Hg(II)-responsive transcriptional regulator [Gammaproteobacteria]	WP_153734274.1	100

			Hg(II)-responsive transcriptional regulator [Oxalobacteraceae]	WP_012079607.1	100
			MerR family transcriptional regulator [*Pollutimonas* sp. M17]	WP_264129062.1	93.62
			MerR family DNA-binding transcriptional regulator [*Pollutimonas* sp. M17]	WP_264130703.1	96.88
	merT	Mercuric ion transporter MerT	Mercuric ion transporter MerT [*Polaromonas* sp. P1(28)-13]	UUZ75192.1	70.63
			Mercuric ion transporter MerT [Oxalobacteraceae]	WP_012079605.1	100
			Mercuric ion transporter MerT [Pseudomonadota]	WP_001294667.1	100
Molybdenum resistance	modA	Molybdate ABC transporter substrate-binding protein	TPA: molybdate ABC transporter substrate-binding protein [Burkholderiaceae bacterium]	HWK69629.1	80.88
	modB	Molybdate ABC transporter permease subunit	TPA: molybdate ABC transporter permease subunit [Burkholderiaceae bacterium]	HWK69630.1	92.14
Cobalt, Nickel resistance	dmeF	CDF family Co(II)/Ni(II) efflux transporter DmeF	CDF family Co(II)/Ni(II) efflux transporter DmeF [*Castellaniella* sp. S9]	WP_269495700.1	88.71
			CDF family Co(II)/Ni(II) efflux transporter DmeF [*Castellaniella* sp. S9]	WP_269495700.1	91.94
			CDF family Co(II)/Ni(II) efflux transporter DmeF [Burkholderiaceae bacterium]	HWK69538.1	95.96
	rcnA	Nickel/cobalt transporter	Nickel/cobalt transporter [*Pusillimonas noertemannii*]	WP_116518094.1	86.21
Cadmium, Lead resistance	cadR	Cd(II)/Pb(II)-responsive transcriptional regulator	Cd(II)/Pb(II)-responsive transcriptional regulator [*Pollutimonas subterranea*]	WP_102072815.1	94.21
			Cd(II)/Pb(II)-responsive transcriptional regulator [*Paracandidimonas lactea*]	WP_255772782.1	92.65
			Cd(II)/Pb(II)-responsive transcriptional regulator [*Pollutimonas* sp. M17]	WP_264131858.1	90.91
Multidrug resistance	acrR	TetR/AcrR family transcriptional regulator	TetR/AcrR family transcriptional regulator [*Pusillimonas* sp. SM2304]	WP_310931984.1	77.46
			TetR/AcrR family transcriptional regulator [*Pollutimonas* sp. M17]	WP_264129616.1	97.37
			TetR/AcrR family transcriptional regulator [*Pusillimonas* sp. MFBS29]	WP_227683018.1	84.88
			TetR/AcrR family transcriptional regulator [*Pollutimonas* sp. M17]	WP_264129980.1	88.32
			TetR/AcrR family transcriptional regulator [*Pollutimonas thiosulfatoxidans*]	WP_128354476.1	75.63
			TetR/AcrR family transcriptional regulator [Alphaproteobacteria bacterium]	TMJ85710.1	47.73
			TetR/AcrR family transcriptional regulator [Burkholderiaceae bacterium]	HWK70546.1	88.83
			TetR/AcrR family transcriptional regulator [Burkholderiaceae bacterium]	HWK69577.1	88.78
	corA	magnesium and cobalt transport protein CorA	Magnesium and cobalt transport protein CorA [*Pollutimonas* sp. M17]	WP_264129550.1	91.32
	czcA		CusA/CzcA family heavy metal efflux RND transporter [Alcaligenaceae]	WP_238665301.1	91.68

		CusA/CzcA family heavy metal efflux RND transporter	CusA/CzcA family heavy metal efflux RND transporter [*Paracandidimonas lactea*]	WP_237174056.1	94.86
	dedA	Metal-sensing transcriptional repressor	Metal-sensing transcriptional repressor [*Pseudomonas*]	WP_013692978.1	83.15
	mdtA	MdtA/MuxA family multidrug efflux RND transporter periplasmic adaptor subunit	MdtA/MuxA family multidrug efflux RND transporter periplasmic adaptor subunit [Burkholderiaceae bacterium]	HWK71840.1	89.83
	sodB	superoxide dismutase [Fe]	Superoxide dismutase [Fe] [*Pollutimonas* sp. M17]	WP_264132116.1	94.79
	sodC	superoxide dismutase [Cu-Zn] SodC	Superoxide dismutase [Cu-Zn] SodC [Burkholderiaceae bacterium]	HWK70841.1	86.05
	tolC	TolC family outer membrane protein	TolC family outer membrane protein [*Pollutimonas* sp. M17]	WP_264129657.1	86.62
			TolC family protein [*Paenalcaligenes niemegkensis]*	WP_238665298.1	97.96
			TolC family protein [*Alcaligenes faecalis*]	WP_321329777.1	80.19
			TolC family outer membrane protein [Burkholderiaceae bacterium]	HWK71820.1	93.72
			TolC family protein [*Pusillimonas*]	WP_123661212.1	84.25
			TolC family protein [*Paracandidimonas lactea*]	WP_237174054.1	91.08
	zupT	ZIP family metal transporter	ZIP family metal transporter [*Pollutimonas* sp. M17]	WP_264130413.1	91.47
Antibiotic resistance	adeC	AdeC/AdeK/OprM family multidrug efflux complex outer membrane factor	Efflux transporter outer membrane subunit [*Pusillimonas* sp.]	WP_325439805.1	74.94

Supplementary Table S5 Cellular fatty acid composition of strain H1-120^T and type strains of other species of the *Pollutimonas* genus. Data for strain H1-120^T were obtained in the present study. Data for the reference strains were taken from previously published descriptions

Strains: 1, *Pollutimonas rozaliensis* H1-120^T (this study); 2, *Pollutimonas subterranea* JR1/69-3-13^T (Babich et al. 2023); 3, *Pollutimonas nitritireducens* JR1/69-2-13^T (Babich et al. 2023); 4, *Pollutimonas thiosulfatoxidans* YE3^T (Koh et al. 2019); 5, *Pollutimonas bauzanensis* BZ59^T (Zhang et al. 2012), 6, *Pollutimonas harenae* B201^T (Park et al. 2011; Li et al. 2020)

	1*	2	3	4	5	6
Cultivation medium used for this analysis	NA2 medium	TSA medium	TSA medium	R2A medium	TSA medium	R2A medium
12:0	5.4	1.6	2.0	7.7	3.8	7.9
16:0	23.9	34.2	34.1	39.2	29.6	32.1
17:0 cyclo	10.7	8.1	6	37.5	24.8	35.7
18:1 *ω*7c	16.6	16.5	13.5	----	8.9	2.2
16:1 *ω*7c	----	32.2	34.5	----	----	----
10:0	TR	----	----	0.5	----	----
12:0 3OH	0.1	0.4	0.5	----	1.8	----
14:1 *ω*5c	0.2	----	----	----	0.1	----
14:0	0.4	0.4	0.6	1.0	0.5	1.7
15:1 *ω*6c	0.8	----	----	----	----	----
15:0	0.6	0.1	0.1	----	----	0.7
16:1 *ω*5c	0.2	----	----	----	0.5	----
17:0	0.7	0.9	----	TR	----	0.4
16:0 3OH	0.1	----	----	----	----	----
18:0	0.5	1.4	----	1.1	0.4	0.6
19:0 cyclo *ω*8c	----	----	----	1.1	0.8	6.0
14:0 3OH	----	1.3	1.1	----	----	----
Iso- 19:0	----	0.6	0.5	0.7	----	----
12:0 2OH	----	----	----	----	2.3	----
10:0 2OH	----	----	----	----	----	----
16:1 2OH	----	----	----	----	----	----
16:0 2OH	----	----	----	----	----	----
Sum in Feature 3 (16:1 *ω*7c/15 iso 2OH)	28.8	----	----	0.6	20.4	1.8
Sum in Feature 2 (14:0 3OH/16:1 ISO I, and/or C12:0 ALDE)	10.3	----	----	9	4.6	8.8

*Data from this study

TR, trace amounts <1%

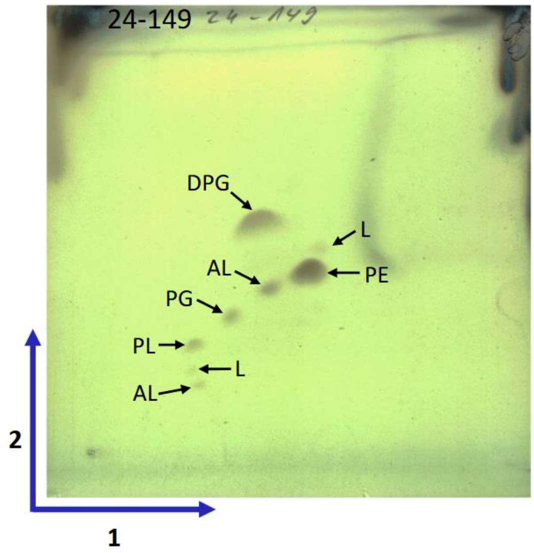

Supplementary Figure S1 Two-dimensional thin-layer chromatography of polar lipids of the H1-120^T strain. The components were visualised using molybdatophosphoric acid, and the specific functional groups were detected using spray reagents that target defined functional groups (Tindall et al. 2007). Abbreviations of the detected polar lipids are as follows: DPG, diphosphatidylglycerol; PE, phosphatidylethanolamine; PG, phosphatidylglycerol; PL, unidentified phospholipid; L, unidentified lipids; AL, unidentified aminolipid
